# Probiotics to improve outcomes of colic in the community: Protocol for the *Baby Biotics* randomised controlled trial

**DOI:** 10.1186/1471-2431-12-135

**Published:** 2012-08-29

**Authors:** Valerie Sung, Harriet Hiscock, Mimi Tang, Fiona K Mensah, Ralf G Heine, Amanda Stock, Elissa York, Ronald G Barr, Melissa Wake

**Affiliations:** 1 Centre for Community Child Health, Royal Children’s Hospital, Parkville, Australia; 2 Murdoch Childrens Research Institute, Parkville, Australia; 3 Department of Paediatrics, University of Melbourne, Parkville, Australia; 4 Department of Allergy and Immunology, Royal Children’s Hospital, Parkville, Australia; 5 Clinical Epidemiology and Biostatistics Unit, Royal Children’s Hospital, Parkville, Australia; 6 Department of Gastroenterology and Clinical Nutrition, Royal Children’s Hospital, Parkville, Australia; 7 Emergency Department, Royal Children’s Hospital, Parkville, Australia; 8 Developmental Neurosciences & Child Health, Child and Family Research Institute, BC Children's Hospital, Vancouver, Canada

**Keywords:** Colic, Crying, Infant, Probiotics, Randomised controlled trial, Health care costs, Postpartum depression, Mental health, Quality of life, Biota

## Abstract

**Background:**

Infant colic, characterised by excessive crying/fussing for no apparent cause, affects up to 20% of infants under three months of age and is a great burden to families, health professionals and the health system. One promising approach to improving its management is the use of oral probiotics. The *Baby Biotics* trial aims to determine whether the probiotic *Lactobacillus reuteri DSM 17938* is effective in reducing crying in infants less than three months old (<13.0 weeks) with infant colic when compared to placebo.

**Methods/Design:**

*Design:* Double-blind, placebo-controlled randomised trial in Melbourne, Australia. *Participants:* 160 breast and formula fed infants less than three months old who present either to clinical or community services and meet Wessel’s criteria of crying and/or fussing. *Intervention:* Oral once-daily *Lactobacillus reuteri* (1x10^8^ cfu) versus placebo for one month. *Primary outcome:* Infant crying/fussing time per 24 hours at one month. *Secondary outcomes:* i) number of episodes of infant crying/fussing per 24 hours and ii) infant sleep duration per 24 hours (at 7, 14, 21, 28 days and 6 months); iii) maternal mental health scores, iv) family functioning scores, v) parent quality adjusted life years scores, and vi) intervention cost-effectiveness (at one and six months); and vii) infant faecal microbiota diversity, viii) infant faecal calprotectin levels and ix) *Eschericia coli* load (at one month only). *Analysis:* Primary and secondary outcomes for the intervention versus control groups will be compared with t tests and non-parametric tests for continuous data and chi squared tests for dichotomous data. Regression models will be used to adjust for potential confounding factors. Intention-to-treat analysis will be applied.

**Discussion:**

An effective, practical and acceptable intervention for infant colic would represent a major clinical advance. Because our trial includes breast *and* formula-fed babies, our results should generalise to most babies with colic. If cost-effective, the intervention’s simplicity is such that it could be widely taken up as a new standard of care in the primary and secondary care sectors.

**Trial Registration:**

Current Controlled Trials ISRCTN95287767

## Background

### The burden of infant colic

Infant colic, characterised by excessive crying/fussing for no apparent cause, is common and distressing to families. Infant colic is defined clinically by Wessel’s criteria of crying and/or fussing ≥3 hours/day for ≥3 days/week for ≥3 weeks [1]. In research, the modified Wessel’s criteria of crying and/or fussing ≥3 hours/day for ≥3 days/week for one week is used for practicality and feasibility, due to the natural course of infant colic symptoms appearing by around three weeks, peaking at eight weeks, and remitting beyond 12 weeks of a term infant’s chronological age. Infant colic affects up to 20% of infants under three months [2,3].

Infant colic poses a great burden to families, health professionals and the health system. Early infant sleeping and crying problems are the most common reason parents seek health professional help in the first three months of life, costing an estimated £65 million pounds per annum in the UK in 2001 [4]. Infant colic has significant adverse effects on maternal mental health and family quality of life [5,6] and is a trigger for child abuse [7,8]. Infants in whom crying persists beyond three months are at risk of adverse outcomes in the school years including anxiety, aggression, hyperactivity, allergy, and sleep disorders [9,10], and at more than double the risk of poor mental health in later years [11]. Families of infants with colic are more dissatisfied with their daily family functioning in later years [12].

After 50 years of research into infant colic, its aetiology remains unclear and evidence-based management options limited. Finding an effective management strategy for infant colic could significantly reduce its associated morbidities and improve the quality of life of many families.

### Proposed aetiological factors for infant colic

Debate continues around whether infant colic represents an extreme of the spectrum of normal crying, or a manifestation of underlying physiological or psychosocial factors. Perhaps, infant colic is best thought of as an exacerbation of infant crying with the aggravation brought about by either physiological factors within the infant, or psychosocial issues with the mother and infant [13]. Proposed psychosocial theories include difficult infant temperament, immaturity in the infant’s ability to modulate reactions to internal and external stimuli, inadequate maternal-infant interaction and maternal anxiety [13]. Physiological theories are based on gastrointestinal factors, including gut dysmotility, excessive intragastrointestinal air, and visceral pain [14,15]. However, no study has been able to definitively prove whether these are causal factors of infant colic. Gastro-oesphageal reflux was traditionally assumed to be a cause of infant distress, but several recent clinical trials have demonstrated that it is an unlikely cause for persistent crying [16,17]. Food allergy is perhaps the strongest factor identified so far in the causal pathway to crying [18-22], with gut inflammation resulting from allergy to cow’s milk protein assumed to be the underlying mechanism. Faecal calprotectin, a gut inflammatory marker, has been identified to be higher in infants with colic compared to those without in one study [23], although another study did not confirm this finding [24]. Another possible mechanism may be an alteration of gut microbiota in infants with cow’s milk protein allergy [25,26].

Recent research has focussed on the pathophysiological role of gut microbiota in the exacerbation of infant crying. This promising new hypothesis is currently generating substantial research interest, with several studies confirming differences in gut microbiota between infants with and without colic. One study indicated increased concentrations of faecal *Clostridium difficile* in infants with colic compared to controls [27], while two studies reported increased *Eschericia coli* (*E coli*) concentrations and reduced *Lactobacillus* species concentrations in infants with colic compared to controls [28,29]. Another study identified certain *Lactobacillus* strains to predominate in infants with colic compared to controls [30], while a more recent study suggested certain *Bifidobacterium* and *Lactobacillus* species to be protective against crying [31]. These findings suggest that while the microbiota are likely to be different in infants with colic, it remains uncertain whether these microbiota differences are the cause or result of the colic condition. Disturbances in gut microbiota may lead to mechanical changes in the gut, such as gas production and bloating and/or gut dysmotility [32,33], which in turn lead to infant crying. The aetiology of infant colic is likely multifactorial.

### Current treatment options for infant colic

Four systematic reviews have evaluated the effectiveness of interventions for infant colic [34-37]. The best evidence supports the use of hypoallergenic or extensively hydrolysed whey or casein-based formula in infants, and instigating maternal elimination diets in breastfeeding mothers, supporting the role of food allergy as a cause of persistent infant crying. However, elimination of cow’s milk from breastfeeding mothers is not always effective and it is unclear which babies respond to hypoallergenic diets and which do not. Other possible effective strategies include improved parental responsiveness, reduced stimulation and the use of sucrose. Ineffective strategies include focused parental counselling, increased carrying, use of car ride stimulators, soy milk and fibre enriched diets. Proton-pump inhibitors for presumed gastrooesophageal reflux disease are ineffective [17]. Simethicone, an anti-foaming agent used to reduce intragastrointestinal gas and bloating, is also ineffective [38,39]. Anticholinergic drugs such as dicyclomine are effective but are associated with significant adverse effects in infants [2,35]. Providing families with support is effective in reducing caregiver stress, but effects on crying are inconclusive [34]. There are therefore no effective and feasible strategies that can be easily implemented to help families with infants with colic.

### Use of probiotics and the potential role of *Lactobacillus reuteri* in infant colic

One promising new approach to the management of infant colic is the use of probiotics. Probiotics are live microorganisms believed to confer health benefits and are used widely in food products, including infant formulae [40]. Probiotics colonise the bowel, competitively inhibit other bacterial adhesion, stimulate host immune responses to pathogens, suppress intestinal inflammation, increase mucus layers and strengthen mucosal barriers [41-43]. Probiotics can modulate infant gut microbiota and increase microbiota diversity [44-52]. A recent study demonstrated specific *Lactobacillus* strains were able to inhibit the growth of gas-producing coliforms isolated in infants with colic [32]. Probiotics and prebiotics have also been shown to alter gastrointestinal motility in newborns by stimulating gastric emptying [33]. Animal studies have shown probiotics to change pain perception mediated by the gut and inhibit gut contractile activity in rats [53-59]. Another explanation is that probiotics may reduce gut inflammation, from whatever cause, in turn reducing associated infant distress.

Four randomised controlled trials have examined the therapeutic effects of probiotics in treating infant colic. In 2007, Savino et al. reported a significant benefit in the use of *Lactobacillus reuteri* (*L reuteri*) strain ATCC 55730 in the treatment of infant colic when compared with simethicone (n = 83) [60]. This study was replicated in 2010 using *L reuteri* strain DSM17938 as the intervention and a placebo (n = 50), with similar promising results [61]. Both studies involved only exclusively breastfed infants whose mothers were all on cow’s milk-free diets. Two other trials using different mixtures of probiotics investigated their therapeutic effects on infant colic (n = 9, n = 62) [52,62] and concluded there were no significant effects on crying time.

The mixed results in the above studies mean that the role of probiotics (including *L reuteri*) in the treatment of infant colic remains uncertain. Moreover, the findings may not necessarily be applicable to the general population since in both the Savino et al. trials, the study populations were restricted to breastfed infants whose mothers were on a cow’s milk-free diet (a select group that is not representative of the general population). Furthermore, there are methodological concerns regarding the previous studies that require further investigation. For example, the comparison group in the 2007 Savino et al. study was simethicone rather than a placebo, which meant that the interventions could not be blinded due to differences in dosage and time of administration. The other trials lacked sufficient power to detect differences and used combinations of probiotics, which can contribute to varying results since probiotic bacteria are known to have species specific effects [52,62]. In summary, there is insufficient evidence to recommend the use of probiotics in infant colic.

### Aims and hypotheses

This double-blind, placebo-controlled randomised trial aims to determine whether the probiotic *L reuteri DSM 17938* benefits infants <3 months old (<13.0 weeks) with colic by reducing daily duration of infant crying/fussing. Secondary aims are whether there is reduced daily frequency of episodes of infant crying/fussing, and improved infant sleep, maternal mental health, and parent and family functioning. We also aim to investigate changes in gut microbiota, faecal calprotectin and *E coli* colonisation which are all possibly implicated in the mechanism of disease, and finally to determine whether probiotic use reduces healthcare costs in infant colic.

We hypothesise that, compared to the placebo (control) group, the *L reuteri* (intervention) group will have lower daily crying/fussing time per 24 hours at one month post randomisation. We further hypothesise that the *L reuteri* (intervention) group at 7, 14, 21, 28 days and 6 months post randomisation will have:

• lower crying/fussing time per 24 hours,

• fewer crying/fussing episodes per 24 hours,

• longer sleep duration,

• higher scores on a standardised measure of maternal mental health (one and six months),

• higher scores on a standardised measure of family functioning (one and six months), and

• higher scores on a standardised measure of parent quality adjusted life years (one and six months).

We also hypothesise that the intervention group compared to the control group will have (at one month):

• more diverse gut microbiota,

• lower faecal calprotectin levels, and

• less *E. coli* colonisation, thereby suggesting a potential pathophysiological mechanism in infant colic.

Finally, we hypothesise that the intervention will be potentially cost-saving to the health care system by diverting families away from more expensive management such as overnight stays at mother-infant units.

## Methods/Design

### Study design, setting and participants

This is a phase III, double-blind, randomised placebo-controlled trial drawing on clinical and community based samples. The trial has been approved by the Royal Children’s Hospital Human Research Ethics Committee (HREC 30111).

Recruitment is from a range of services widely used by and readily accessible to parents seeking medical advice regarding their crying babies in Melbourne, Australia. These comprise: the Royal Children’s Hospital Emergency Department (RCH ED), the RCH Unsettled Babies clinic (a tertiary referral-based clinic for assessment and management of unsettled babies), Tweddle Child and Family Health Centre (a mother-infant parenting centre), two Maternal Child Health centres (universal nurse health checks in the Boroondara and Moonee Valley districts), and paediatricians at the RCH and in private practices. Interested families can also directly contact the study team to be involved.

A total sample of 160 infants less than three months (13.0 weeks) old with infant colic is being recruited between August 2011 and August 2012. The expected total duration of the study from the start of recruitment to the last subject finishing the six month follow-up is 18 months. The treatment period is one month, with a follow-up period of six months.

### Inclusion criteria

Each infant must meet all of the following criteria to be enrolled in this study:

1. Infant colic, i.e. crying or fussing ≥ 3 hours/day for ≥ 3 days over seven days (as defined by the modified Wessel’s criteria) by caregiver’s report at the time of study commencement;

2. Less than three months (i.e. up to and excluding 13.0 weeks) old at the time of study commencement;

3. Greater than 36 weeks gestation at birth; and

4. Birth weight of more than 2500 g.

### Exclusion criteria

Infants meeting any of the following criteria are excluded from the study:

1. Failure to thrive (weight gain < 100 grams/week averaged from birth to the last recorded weight);

2. Major medical problems (eg. ill, immunocompromised, major developmental or genetic abnormality);

3. Taking solids, antibiotics or *L reuteri* and, if breastfeeding, has mother taking *L reuteri* at the time of study commencement;

4. Cow’s milk protein allergy, defined as resolution of crying after a paediatrician-recommended two week trial of dairy-free diet in the infant, or in the mother if breast-feeding, at the time of study enrolment; and

5. Caregiver has insufficient English to understand informed consent and complete questionnaires.

### Randomisation

A block randomisation schedule to maintain balance between treatment arms has been prepared by an independent statistician, not directly involved in the analysis of the study results, from the Clinical Epidemiology and Biostatistics Unit (CEBU) at the RCH and supplied to Pharmacy who dispense the study product. Randomisation is stratified by method of infant feeding (breastfed versus formula-fed) and age at the time of study commencement (≤6 weeks versus >6 weeks, due to the natural crying peak occurring at around six weeks of age). Infants on a mixture of both breast- and formula-feeding are allocated to the formula-fed stratum. To minimise potential biases, the study is double-blind whereby treatment allocation is concealed from all study investigators and participants.

### Treatment arms, dosage and route of administration

The intervention is *L reuteri DSM 17938* (0.2x10^8^ cfu per drop as five drops per day) in an oil suspension. Under refrigeration, it is stable for 21 months at 2°C to 8°C (as documented by the manufacturer, BioGaia AB, Stockholm, Sweden). The placebo is maltodextrose in the same oil suspension, identically packaged and stored, and has the same appearance, colour and taste as the intervention. Both intervention and placebo are labeled with only the randomisation number, batch number, expiry date, and the statement “For clinical trial use only”.

Carers administer five drops of study product orally to each infant once daily for 28 days. The dose does not need to be given at a fixed time each day, nor does it need to be given with feeds. However, for compliance and ease of administration, we recommend that families give the dose with the same feed each day.

### Outcome measures

Table 1 shows the primary and secondary outcome measures at different time points of the study. Table 2 shows other information collected at different time points, including family demographics, potential confounders, physical assessments, and records of compliance and side effects.

**Table 1:** Outcome measures

**Construct**	**Timing (D = days/M = months)**						**Measure**	**Additional Information**
	**0**	**D7**	**D14**	**D21**	**D28**	**M6**		
**Primary Outcome**								
Infant crying/fussing time (mins/day)					■		Study diary	A validated measure of infant crying/fussing/sleep/feeding and records these behaviours in 5 minute epochs over 24 hours [65-67]. At baseline parents record in the study diary for 24 hours. At other time-points, the diary is filled in over 48 hours. This is to account for daily variability in infant behaviour whilst taking into account the potential burden to families from filling in the diary for prolonged periods of time.
**Secondary Outcomes**								
Infant crying/fussing time (mins/day)	■	■	■	■		■	Study diary	
Number of episodes of crying/fussing/day	■	■	■	■	■	■	Study diary	
Infant sleep duration (mins/day)	■	■	■	■	■	■	Study diary	
Maternal mental health scores	■				■	■	Edinburgh Postnatal Depression Subscale (EPDS)	A 10-item validated questionnaire to screen for depression in the post-partum setting, with higher scores indicating poorer mental health [68]. Scores of ≥10 and ≥12 are validated to detect postnatal depression in community and clinical settings, respectively.
Parent functioning scores	■				■	■	PedsQL Family Impact Subscale	A 5-item validated questionnaire to assess family functioning, with higher scores indicating better family functioning [69].
Infant functioning scores						■	PedsQL Infant Subscale	A 36-item validated questionnaire used to measure infant physical, emotional, social and cognitive functioning [70].
Parent quality adjusted life years scores	■				■	■	AQol-4R	A 12-item validated questionnaire to assess the health economic parent quality of life [71].
Health service use	■				■	■		Additional health professional visits in relation to infant’s crying.
Infant faecal microbiota diversity	■				■		16SrDNA amplification (T-RFLP)	A molecular method to investigate the diversity within bacterial communities, given as a diversity score, with higher scores indicating more microbial diversity [72].
Infant faecal calprotectin (mg/kg)	■				■		ELISA	An enzyme-linked immunosorbent assay to detect the presence of calprotectin, a marker of gut inflammation [73].
Infant faecal *E coli* load (cfu/ml)	■				■		Quantitative PCR	A molecular method to detect and measure the presence of particular marker genes of *E coli*[74].

**Table 2:** Additional information collected

**Measure**	**Timing (D = days/M = months)**						**Information**	**Details**
	**0**	**D7**	**D14**	**D21**	**D28**	**M6**		
Questionnaire	■						Demographics	
Questionnaire	■						Potential confounders	Family history of atopy. Antenatal / current probiotic / antibiotic use. Smoking during pregnancy. Mode of delivery (caesarean versus vaginal).
Diary questions	■	■	■	■	■	■	Potential confounders	Infant feeding method (breast versus formula). Mother’s intake of dairy, probiotics, medications. Infant’s intake of dairy, probiotics, solids, medications. Infant gastro-oesophageal reflux symptoms (measured by the Infant Gastroesophageal Reflux Questionnaire Revised I-GERQ-R [75,76], a validated measure of infant gastro-oesophageal reflux). Settling techniques. Concurrent illnesses / immunisations.
Diary questions	■	■	■	■	■		Compliance	Number of days study drops missed over preceding week.
Diary questions	■	■	■	■	■		Side effects	Infant stool frequency, consistency.
Physical examination	■						To exclude organic causes of crying	Infants recruited through Maternal Child Health Nurses and Tweddle are examined by the study paediatrician.
Weight	■				■		Weight (kg, to nearest gram)	Measured by the Wedderburn Infant Scale (Tanita Baby Scale Model BD590) calibrated for the study.
Infant faecal *L reuteri* (cfu/ml)	■				■		Quantitative PCR	A molecular method to detect and measure the presence of particular marker genes of *L reuteri*, as a measure of compliance [77].

### Study procedure details

**Figure 1:**
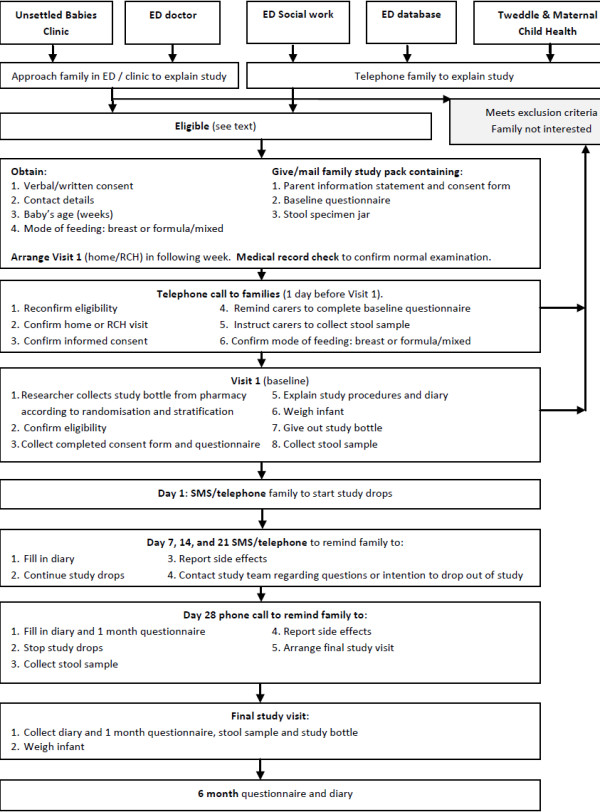
Study procedures.

**Figure 2:**
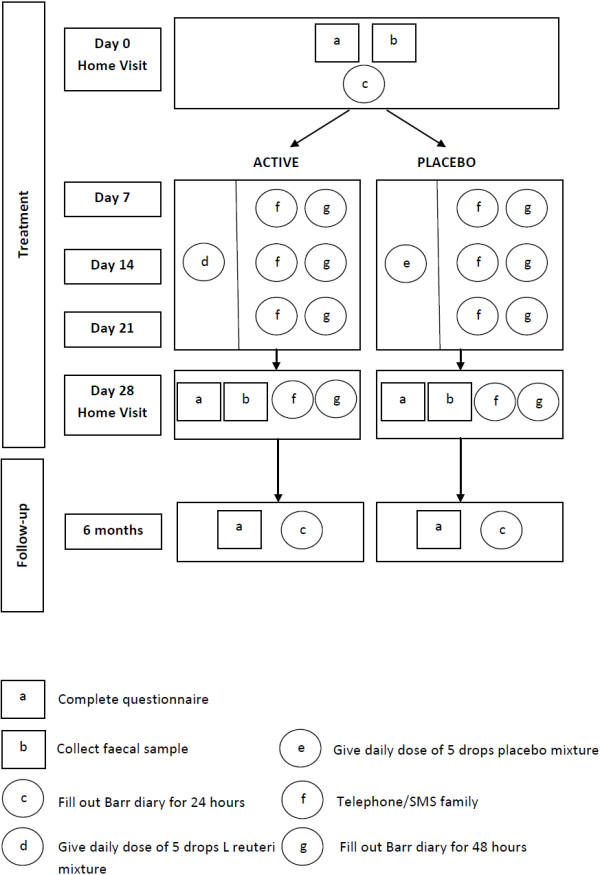
Perera diagram showing treatment and follow-up procedures.

Figure 1 and Figure 2 show details of study procedures.

#### Recruitment

Eligible families who present through the RCH ED are identified in three ways: i) by the treating doctor, ii) by the ED social worker, or iii) through the ED electronic database. Eligible families who present through the RCH Unsettled Babies Clinic are identified by their treating doctors. Eligible families who present through the Boroondara and Moonee Valley Maternal and Child Health Nurses and Tweddle Child and Family Services are identified by their nurses or intake worker. Potentially eligible families are referred to study team members, who call families or approach them in the ED or clinic if appropriate.

The study team establishes eligibility criteria. Infants and breastfeeding mothers who are on a paediatrician-recommended dairy-free diet, infants who are taking *L reuteri*/solids/antibiotics, and breastfeeding mothers who are taking *L reuteri* at the time of study enrolment are reassessed for eligibility by telephone call after two weeks. Eligible families are approached for consent. Each family can choose to be visited at home or attend a visit at RCH for the first study visit. Study researchers call the families one day before the proposed study visit to confirm the home or RCH visit. During this call, study researchers reconfirm eligibility and consent, remind families who have not completed the consent form and baseline questionnaire to complete them, and instruct caregivers to collect a stool sample from their babies and store it in a freezer. Study researchers also record on the Clinical Record Form whether the babies are breast- or bottle-fed.

#### Visit one

During the visit, the researcher reconfirms eligibility, asks the mother to complete the baseline questionnaire, and reviews with the family in detail the study diary, anticipated phone calls, the procedure of administering the study product to the infant, and the anticipated procedures at the end of the study. The researcher weighs the baby, collects the stool sample and transports it back to the RCH on ice in a portable cooler, and then stores it in the laboratory −80°C freezer.

#### Study period Days 1 – 28

On days 1, 7, 14 and 21 the study team sends a mobile text (SMS) message to families to remind families to fill in the weekly diary. Study researchers call families on day 28 to remind families to a) stop administration of the study product; b) fill in the one month questionnaire; c) collect a stool sample; and d) make an appointment for the second (final) visit.

#### Visit two

During the second visit, the researcher weighs the infant, and collects a) the study diary; b) the one month questionnaire; c) the study product bottle and d) a stool sample which is transported back to the RCH on ice in a portable cooler, and then stored in the laboratory -80°C freezer.

#### Six month follow-up

At six months, the families are sent a letter together with a 24-hour study diary, a questionnaire and a reply-paid envelope. Caregivers are asked to complete the study diary and questionnaire, and return both in the enclosed reply-paid envelope.

#### Compliance with the study product

Compliance is assessed by weighing of study bottles pre- and post- dispensing, collection of faecal samples at the end of the intervention period to assess for presence of *L reuteri* in each infant’s stool, and weekly diary to record administering of the product.

### Data analysis

#### Estimation of sample size

We have selected a sample size of 160 to provide 80% power to detect a minimum effect size of 0.5 standard deviations, difference in the mean daily crying time between treatment groups with a significance level of p < 0.05, allowing for a drop out rate of 20%. If the data are substantially skewed, 80% power would be maintained to detect a minimum effect size of 0.525 [63,64]. In Savino’s trial [60], the percentage reduction in median daily crying time at day 28 compared to baseline was 70% for the probiotic and 26% for simethicone, suggesting substantive differences in this outcome are likely. With a much larger sample size (double that of Savino’s trial) in our study, we are confident we would also be able to detect a smaller difference in daily crying time reduction. Our sample size of 160 infants provides 80% power to detect a minimum effect size of 0.85 standard deviations, difference in the mean daily crying time between treatment groups within either the formula-fed or breastfed babies, assuming 40% of infants in the sample are exclusively breastfed.

We will be conducting an intention-to-treat analysis in which participants are compared according to the group to which they were randomly allocated regardless of participants' compliance, crossover to other treatments or withdrawal from the study. This approach preserves the prognostic balance in the study arms achieved by randomisation.

Baseline characteristics and study outcomes will be described for each treatment group using means and standard deviations for normally distributed continuous outcomes, while medians and inter-quartile ranges will be used for continuous outcomes that are skewed. Proportions for categorical data will be given. All primary and secondary outcomes for the intervention versus control groups will be compared with t tests and non-parametric tests for continuous data, and chi squared tests for dichotomous data. The primary outcome is the crying time/24 hours at day 28. We will additionally consider a dichotomised indicator of treatment response defined as a 50% reduction in crying time. Subgroup analyses are intended a priori to examine treatment differences amongst breastfed babies and those who are formula-fed, and amongst infants with a family history of atopy and those without.

Regression models will be used to estimate treatment effect sizes, odds ratios and 95% confidence interval, adjusting for potential confounding factors identified a priori and measured at baseline. These include infant age (≤6 weeks versus >6 weeks, taking into account the natural course of infant colic symptomatology with the peak of crying at six weeks), mode of birth delivery, maternal smoking, use of antibiotics or probiotics in infants and breastfeeding mothers, maternal diet if breastfeeding, maternal mental health scores, and known causes of crying (e.g. fever, eczema, vaccination, vomiting, and diarrhoea). Random effects regression models will be used for further longitudinal analysis examining trends in crying time within individual babies.

## Discussion

Infant colic is common, distressing, impacts adversely on maternal mental health, and is a risk factor for shaken baby syndrome. An effective, practical and acceptable intervention for infant colic would represent a major clinical and public health advance. As our trial will include breast *and* formula-fed babies, regardless of maternal diet, our results can be generalised to most babies presenting with colic. If cost-effective, the simplicity of the intervention is such that it could be widely taken up as a new standard of care in the primary and secondary care sectors.

With the boom of probiotic products appearing on the market, the use of probiotics in the community is becoming more widespread. It is therefore important to provide sufficient sound scientific evidence for its effectiveness in infant colic before it becomes incorporated into regular use by the community, and avoid unnecessary consumption of costly products if the intervention is not found to be effective.

## Abbreviations

*L reuteri*: *Lactobacillus reuteri*; *E coli*: *Eschericia coli*; RCH: Royal Children’s Hospital; ED: Emergency Department; CEBU: Clinical Epidemiology and Biostatistics Unit; cfu: Colony forming units; SMS: Short message service; mins: Minutes; ml: Milliliter; kg: Kilogram; mg: Milligram.

## Competing interests

All authors declare that they and their spouses, partners or children have no financial and non-financial relationships or interests that may be relevant to the submitted work. The authors declare they have no competing interests.

## Authors’ contributions

VS, HH and MW conceived the *Baby Biotics* trial with MT, RH and FM. VS is the Project Manager and takes overall responsibility for all aspects of the trial and this manuscript. EY is the Research Assistant. VS, HH and MW designed the intervention, with advice from MT, RH and RGB, who also advised on measures and their interpretation. FM advised on statistical issues. AS contributed to recruitment procedures. All authors contributed to, read and approved the final manuscript.

## Authors’ information

VS is a paediatrician and is undertaking a PhD in the area of infant colic. HH and MW are her supervisors.

## Pre-publication history

The pre-publication history for this paper can be accessed here:

http://www.biomedcentral.com/1471-2431/12/135/prepub
